# Parasitological and Molecular Observations on a Little Family Outbreak of Human Fasciolosis Diagnosed in Italy

**DOI:** 10.1155/2014/417159

**Published:** 2014-03-04

**Authors:** Simona Gabrielli, Pietro Calderini, Luigi Dall'Oglio, De Angelis Paola, De Angelis Maurizio, Scottoni Federico, Gabriella Cancrini

**Affiliations:** ^1^Umberto I Policlinic of Rome, Viale del Policlinico 155, 00161 Rome, Italy; ^2^Department of Public Health and Infectious Diseases, Sapienza University, Piazzale Aldo Moro 5, 00185 Rome, Italy; ^3^Experimental Zooprophylactic Institute, Via Tancia 21, 02100 Rieti, Italy; ^4^Bambino Gesù Children's Hospital, Piazza Sant'Onofrio, 4, 00165 Rome, Italy

## Abstract

In the year 2010, three children who were born in a Romanian cattle farmer family went to Italy to join their mother. One of them was admitted to an Italian pediatric hospital for severe anemia that, when she was in her country, had been treated with blood transfusion. Blood tests and an abdominal ultrasound study triggered the suspicion of biliary parasitosis. The child underwent a cholangiopancreatography that caused the release of parasitic material microscopically identified as *Fasciola hepatica*. All children and their mother were submitted to coproparasitological analyses, which identified *F. hepatica* eggs only in the patient and in her twin sister. Parasitic materials recovered and flatworm specimens by us *ad hoc* obtained from Italian and Romanian cattle were genetically (*ITS* and *COI* genes) analyzed, and their sequences were compared with those deposited in GenBank. Specimens from children clustered with the Romanian strain examined and showed remarkable genetic differences with flatworm specimens from Italy. Anamnesis, parasite biology, and genetic data strongly suggest that twin sisters became infected in Romania; however, human fasciolosis is an emerging sanitary problem, favored by climate changes and global drivers; therefore, it deserves more attention on behalf of physicians working in both developing and developed countries.

## 1. Introduction

Fasciolosis is a major veterinary health problem of herbivorous such as cattle, sheep, and goats worldwide due to the economic losses it causes in animal husbandry, whereas human infection has been considered, up to 1990, a disease of secondary relevance [1]. In last decades, zoonotic fasciolosis emerged or reemerged in more than 60 countries, including Italy [2, 3]. Prior to 1992, total reported cases of human infections were estimated to be less than 3,000, but recent conservative estimates on its burden indicate that the number of individuals infected worldwide is at least 2.65 million [4]. Emergence, long-term pathogenicity, and immunological interactions [5, 6] prompted the WHO to include human fasciolosis on the list of priorities among the so-called neglected tropical diseases (NTDs) [7], which are chronic, debilitating, poverty-promoting, and among the most common causes of illness in developing countries.

The emergence seems to be partly related to climate changes and so-called global drivers, among which mainly anthropogenic modifications of the environment and increasing short- and long-distance travels and import/export facilities available nowadays. At present, it is the “at indirect life-cycle” parasitic disease presenting the widest known latitudinal, longitudinal, and altitudinal distribution [8]. Indeed, *Fasciola hepatica* expanded from its European original geographical area and colonized five continents.

The aims of this paper are to report parasitological observations on a family outbreak of human fasciolosis diagnosed in Italy and to molecularly characterize the parasites recovered.

## 2. Subjects and Methods

### 2.1. Medical History

Two nine-year-old Caucasian female children, born in Romania where they far back suffered from unspecified anemia, in 2010 went to Italy (Perugia city) together with their sister aged 6 to join their mother. The persistent severe microcytic hypochromic anemia of one of them, who in Romania even had required a blood transfusion, urged the mother to submit her to medical controls at the “Bambino Gesù” Children's Hospital in Rome. Preliminary blood tests were performed and noticed, besides normal bilirubinaemia and slightly elevated values for liver and pancreatic enzymes, anemia (Hb: 9.8 gr/dL) and eosinophilia (22.2%). An abdominal ultrasound study was carried out, which triggered the suspicion of biliary parasitosis. Therefore, an endoscopic retrograde cholangiopancreatography was started, whose initial precut sphincterotomy caused the release of both abundant grey gelatinous material mixed with bile and an active parasite that was gathered.

On the basis of arrangements made with the “Sapienza” University, the parasitic material was sent to the Laboratory of Parasitology. In addition, on both the patient, her sisters (recently arrived in Italy), and their mother (working in Italy since 2004) further parasitological analyses were planned, as well as the collection of data about the family lifestyle.

The parasitic material gathered from the patient and 3 stool samples from each family member were submitted to macro- and microscopic examinations and to molecular investigations (DNA extraction, polymerase chain reaction (PCR) amplification, and sequencing analysis).

### 2.2. Parasitological Analyses

The parasitic material gathered was repeatedly washed in physiological saline solution, macroscopically examined and measured, explored by microscopy, and morphologically identified according to morphological keys [9]. Then, it was fixed in 70% ethanol until genomic DNA extraction.

As for stool samples, 250 mg was stored for molecular analyses, whereas 5 g was immediately submitted to both direct and after Ridley concentration microscopic analyses [10] and to the evaluation of the parasitic burden (eggs per gram of stool) (epg).

### 2.3. DNA Extraction and PCR Amplification

Genomic DNA was extracted from 25 mg adult specimen using the NucleoSpin tissue kit (Macherey-Nagel, Duren, Germany). Total DNA was extracted from stools of each subject by QIA amp DNA stool mini kit (Qiagen, Hilden, Germany), pretreating the samples according to the manufacturer's instructions. Briefly, 180–200 mg of each fecal material was transferred into an Eppendorf tube and dissolved in 700 *μ*L of ASL buffer of DNA extraction kit. Samples were then exposed to five cycles of freeze and thaw within liquid nitrogen and boiling water; afterwards, ASL buffer (700 *μ*L) was added into each tube. The DNA extraction was carried out by following the kit instructions.

PCR amplification was performed in 25 *μ*L volumes under the following final conditions: 1x buffer including 1.5 mM MgCl_2_, 0.2 mM of each dNTP, 1 *μ*M each of forward and reverse primers, and 1 unit of polymerase (BIOTAQ DNA Polymerase, Aurogene, Rome, Italy). The negative control was a reaction mixture in which the DNA template was replaced by distilled water. Positive controls were DNAs extracted from 4 *F. hepatica* specimens *ad hoc *gathered from a cow slaughtered in Central Italy and 3 specimens collected from a cow imported from Romania (FhCentrIt and FhRom, resp.). Genomic DNA of positive controls was obtained by applying the procedure for DNA extraction from tissue described above.

The DNA region (about 1,000 bp) comprising *ITS*-1, 5.8S rDNA, and *ITS*-2 was amplified by polymerase chain reaction using primers BD1 (forward: 5′-GTCGTAACAAGGTTTCCGTA-3′) and BD2 (reverse: 5′-TATGCTTAAATTCAGCGGGT-3′) [11, 12]. Two conserved primers, Ita8 (forward: 5′-ACGTTGGATCATAAGCGTGT-3′) and Ita9 (reverse: 5′-CCTCATCCAACATAACCTCT-3′) [13], were used to amplify 439 bp of the *COI* gene following the protocol previously published [14].

### 2.4. DNA Sequence Analysis

PCR products from children and cows (FhCentrIt and FhRom) were purified using the SureClean kit (Aurogene, Rome, Italy), following the manufacturer's instructions, and were directly sequenced with PCR primers in both directions by an external sequencing core service (Eurofins MWG Operon, Anzinger, DE). Sequences obtained were corrected by visual analysis of the electropherograms; then, they were aligned with relevant *ITS* and *COI* sequences previously published [14] (Table 1) to evaluate intraspecific divergences for each marker and to construct a phylogenetic tree of *COI* sequences (neighbor-joining method, 1000 bootstrap replicates) (MEGA v5.0 software package) [15].

## 3. Results

**Figure 1:**
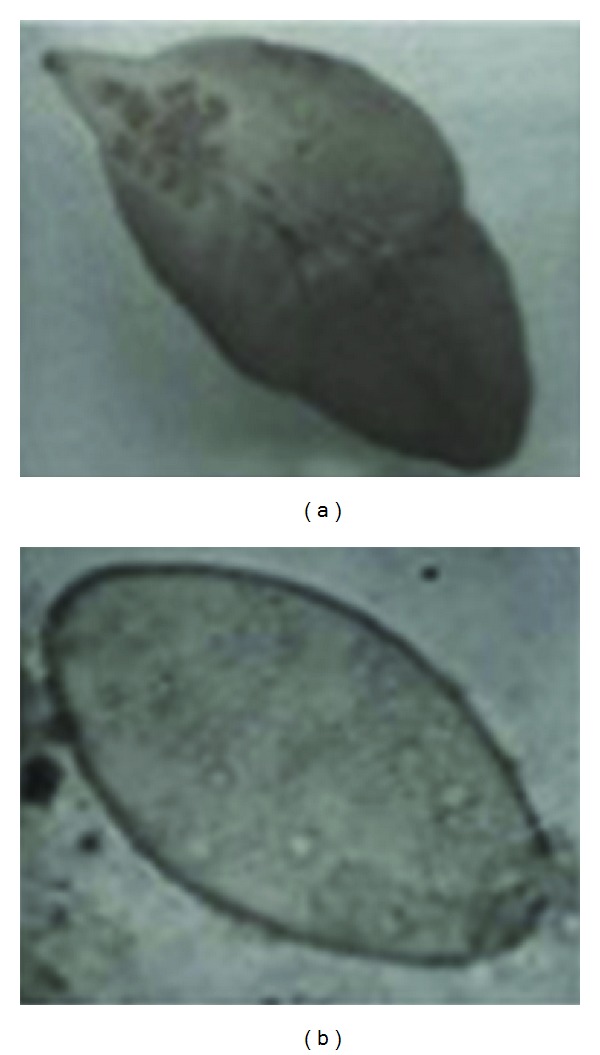
(a) The flat, brownish leaf-shaped adult specimen (2.5 × 1 cm) gathered from the patient; (b) operculated egg of *Fasciola hepatica* (140 × 80 *μ*m) evidenced in stool samples by microscopy.

Macroscopic and microscopic analyses of the surgically removed material evidenced, besides fragments of other damaged specimens, a flat, brownish leaf-shaped organism measuring 2.5 × 1 cm, with a cone-shaped projection followed by a body enlargement, a spiny tegument, and two powerful suckers (Figure 1(a)). It was identified as *F. hepatica*, confirming the endoscopic suspicion. Only 2/4 examined subjects proved positive to coproparasitological analyses: the twin sisters. Stool samples of the child submitted to the endoscopic retrograde cholangiopancreatography evidenced a mean number of 85 epg of *F. hepatica* and that of her twin sister, who also proved positive to radiological analyses immediately performed, 50 epg. On the basis of their shape and mean size (140 × 78 *μ*m), the operculated eggs were identified as *F. hepatica* eggs (Figure 1(b)). Further 8 coproparasitological controls were performed on the third child and her mother proved to date to be negative.

**Figure 2:**
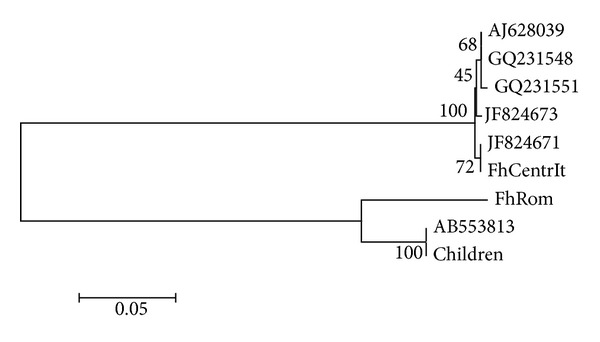
Dendrogram obtained by means of neighbour-joining analysis of *COI* sequences. Numbers at the nodes indicate percentage of bootstrap support obtained in 1,000 replicates. The scale bar indicates the p-distance of the branches.

Molecular diagnostics confirmed all parasitic material from the sisters as belonging to *F. hepatica*. Sequences of the *ITS* yielded high identity with *F. hepatica* specimens collected from Spain, Italy, Turkey, Algeria, and Romania (p-distance from 0.000 to 0.011). As expected, analysis of the *COI* gene evidenced more intraspecific variability than *ITS* analysis. In detail, specimens from children showed a close relationship with *F. hepatica* from Egypt, clustered with the Romanian strain now examined (FhRom), and evidenced remarkable genetic differences if compared with flatworm specimens from Italy (FhCentrIt), France, and Tunisia (p-distance from 0.000 to 0.482), all clustering in a distinct group (Figure 2).

## 4. Discussion

Notwithstanding this zoonosis is mainly evidenced in developing countries, in the last decades, the number of cases detected in developed ones has increased. However, being not subject to mandatory notification, the estimation of prevalence can only be based on some reports and a retrospective analysis of data from laboratories of health centres or hospitals [2, 16–18].

In Italy, at least 7 human cases have been described [19–22] and about 10 cases/year are diagnosed [3]. Few and dated reports are available for Romania [23–25].

This little outbreak of human fasciolosis has been diagnosed in Italy. However, even if the liver fluke is present in most Italian territories, Central Italy included, making the infection risk for children possible in Italy as in Romania, we believe these infections did not originate in Italy. Indeed, the anamnesis and the parasite biology first of all suggest that this family outbreak is not autochthonous. We know that people get infected by eating uncooked aquatic plants or vegetables grown in sheep/cattle-raising rural areas where cercariae are released by the intermediate host snails and excyst as metacercariae. When people drink/eat uncooked infected abovementioned materials, infective metacercariae excyst in the duodenum, larvae emerge and penetrate the wall of the small intestine and the liver capsule; then they pass through the liver tissue into the biliary tract, where they grow into hermaphroditic and haematophagous adult worms. These crossings are favored by the spiny tegument, also at the origin of the traumatic destruction of the liver parenchyma followed by the inflammatory cell infiltration and fibrosis. The migration of large numbers of larvae can cause abdominal pain, biliary colic, fever, hepatomegaly, elevated liver enzymes, and eosinophilia, and, in children, anemia and even death. Prepatent period is long (about 3 months), but longer times are necessary to develop severe anemia.

In the light of the parasite biology and considering that the two sisters, born in a cattle farmer Romanian family, spent most of their life in a pasture environment (Buzau, NE of Bucharest, close to a river that floods into Danube) and in precarious hygienic conditions, entrusted to their old grandmother, we strongly suppose that they get infected in their country. Indeed, in Italy they lived in urban areas (Perugia city) and spent in our country only few months before the fasciolosis detection. Such period is enough for the parasite development but it is too short to develop clinical manifestations up to severe anemia, a problem that had been evidenced a long time ago, and, when they stayed in Romania, a blood transfusion for one of the sisters was required.

Genetic analyses that we performed to enforce our belief about the origin of the infection did not yield decisive results, at least at the first analysis. As previously described, internal transcribed spacers *ITS*-1 and *ITS*-2 useful to discriminate between *F. hepatica* and *F. gigantica *[12] showed a very low intraspecific variability and confirmed to be unrelated to geographical origin of the specimens or to the host [26]. On the contrary, the *COI* analysis evidenced that *F. hepatica* isolated in these two cases of human infection differs from all Italian isolates and it is more related to the Romanian isolate.

## 5. Conclusion

Even if we cannot definitively exclude a possible Italian origin of the infection, anamnesis, parasite biology, and genetic analyses strongly suggest that both patients became infected with *F. hepatica* in their origin country. However, fasciolosis, almost anywhere present in animals, is a neglected zoonosis that should be carefully considered by physicians in any country, since climate changes and global drivers are favoring its emergence.

## Figures and Tables

**Table 1:** *ITS* and *COI* sequences of *Fasciola hepatica* used to perform the multiple sequence alignment.

*ITS*		*COI*	
Collection site	Accession number	Collection site	Accession number
Spain	AM709649	France	AJ628039
Spain	AM709621	Tunisia	GQ231548
Turkey	FJ593632	Tunisia	GQ231551
Algeria	GQ231547	Italy	JF824673
Italy	JF824668	Italy	JF824671
Italy	JF824669	Central Italy	Not deposited
Central Italy	Not deposited	Romania	Not deposited
Romania	Not deposited	Egypt	AB553813
